# How do they eat: a digital diet ethnography of dietary behavior determinants among New Zealand and Chinese university students

**DOI:** 10.3389/fnut.2025.1729437

**Published:** 2026-01-12

**Authors:** Xingbo Li, Nick Ariell, Andrea Braakhuis, Zengning Li, Rajshri Roy

**Affiliations:** 1 Faculty of Health and Medical Sciences, The University of Auckland, Auckland, New Zealand; 2 The First Hospital of Hebei Medical University, Shijiazhuang, Hebei, China; 3 Hebei Province Key Laboratory of Nutrition and Health SZX2021021, Shijiazhuang, Hebei, China; 4 Charles Perkins Centre, The University of Sydney, Sydney, NSW, Australia; 5 Nutrition and Dietetics, Sydney School of Nursing, Faculty of Medicine and Health, The University of Sydney, Sydney, NSW, Australia

**Keywords:** dietary behavior, digital ethnography, food availability and accessibility, food environment, university student

## Abstract

**Background:**

Non-communicable diseases (NCDs) are a leading cause of global mortality and poor dietary habits are key contributors. University students are especially vulnerable to obesogenic food environments, yet cross-cultural qualitative evidence on how campus and home food environments shape student dietary behaviors is limited. This study used digital diet ethnography to compare determinants of dietary behavior among university students in New Zealand and China.

**Methods:**

Nine New Zealand and ten Chinese university students recorded their meals and contextual reflections via the Indeemo platform for 90 days. Multimodal data (photos, videos, text) were thematically analyzed using a hybrid inductive–abductive approach informed by cohort-specific theoretical frameworks; coding followed a harmonized content-analysis protocol with double-coding, consensus meetings, and manual verification of automated transcripts.

**Results:**

New Zealand findings coalesced into four themes: (1) time is of the essence, (2) cash is king, (3) the on-off campus conundrum, and (4) miscellaneous influences. In China three major themes emerged: (1) social and environmental determinants, (2) food-related determinants, and (3) intrapersonal determinants. Clear distinctions exist between on-campus and at-home datasets. Across cohorts, time scarcity, perceived cost, convenience, sensory preferences and social influences were dominant drivers. However, their mechanisms differed according to institutional food systems (marketized options vs. subsidized canteens) and cultural contexts.

**Conclusions:**

University students' suboptimal dietary patterns primarily reflect rational, pragmatic responses to shared contextual constraints rather than individual deficits in diet behaviors. The consistency of these constraints across divergent food environments underscores the need for structural interventions that increase the availability, affordability, convenience, and sensory appeal of healthier options while leveraging each system's strengths to make healthy eating the default choice.

## 1 Introduction

Non-communicable diseases (NCDs) account for 41 million deaths annually, which equates to 74% of all global deaths (1). Unhealthy diet is listed as one of the major factors that significantly increases NCD risk (2). Despite global nutrition education and health campaigns, obesity remains a significant public health issue in many countries (3–5). Researchers are seeking to understand how the physical and digital food environment plays a role in the prevention and control of obesity in diverse settings (6–8). Young adults (18–24 years) constitute the majority of tertiary education population and are susceptible to weight gain when they are frequently exposed to an obesogenic food environment (9). The university food environment influences student dietary behaviors from multiple perspectives, including taste, price, and accessibility (10). To build on previous findings, qualitative evidence is critical for studying the relationship between the food environment and university student dietary behaviors (11–13). With obesity prevalence rising, there is a significant paucity of evidence around student dietary behaviors within the context of New Zealand and Chinese universities (14–18). Of the studies that have assessed student dietary behavior determinants in Western university settings, most focused on the barriers and enablers of healthy eating (19–21). The food environment in New Zealand universities is characterized by a high prevalence of unhealthy, energy-dense, and nutrient-poor foods, with healthy options often less available, less accessible, and more expensive (10). Only a small proportion of campus food outlets can be classified as healthy, and students frequently purchase food on campus, with taste, price, and value for money being key determinants of choice (22). The dominance of unhealthy foods is consistent with global trends in university settings (23–26). Unlike many Western contexts where university food services increasingly emphasize health promotion and sustainability, Chinese university food service guidelines primarily frame food provision as a public welfare responsibility, prioritizing affordability and safety over nutritional quality or environmental considerations (24). Published investigations into the Chinese on food environment are scarce, but are largely concentrated on operational aspects such as food safety, hygiene standards, and the mitigation of food waste, with comparatively limited attention to dietary diversity, health outcomes, or policy-driven nutrition interventions (25–27).

Food policy, through regulations, standards, and institutional governance, plays a critical role in shaping campus food environments by influencing what foods are available, affordable, and promoted (28). In New Zealand, university food policies increasingly align with public health objectives, incorporating nutrition guidelines and sustainability principles, although implementation remains fragmented. Conversely, Chinese university food service guidelines primarily emphasize affordability and food safety as part of their public welfare mandate, with limited attention to nutrition quality or environmental impact (29). Across both contexts, existing policies often address single issues such as waste reduction or procurement rather than adopting a holistic approach that integrates health and sustainability (30). Economic factors further constrain dietary choices: rising food costs and limited student budgets in both countries push students toward cheaper, energy-dense options, reducing dietary diversity and heightening food insecurity (23). These financial pressures also hinder the adoption of sustainable diets, as healthier and environmentally friendly foods are frequently perceived as less affordable or accessible (31). Promoting sustainable and nutritious diets in universities therefore requires multi-level strategies, including policy reform, education initiatives, and environmental changes that make healthy, low-impact foods both available and appealing (32).

In this explorative study, we use digital diet ethnography to elicit qualitative insights and add nuance to the “how and why” of university student dietary behaviors (33, 34). We aim to understand how external and internal dietary behavior determinants, including the physical food environment and the university lifestyle, influence New Zealand and Chinese university students. We aim to synthesize critical determinants with the aid of comparison and contrast between ethnography findings under two cultural settings. Potential implication of key determinants on university food environment intervention is discussed.

## 2 Methods

### 2.1 Overview

**Figure 1:**
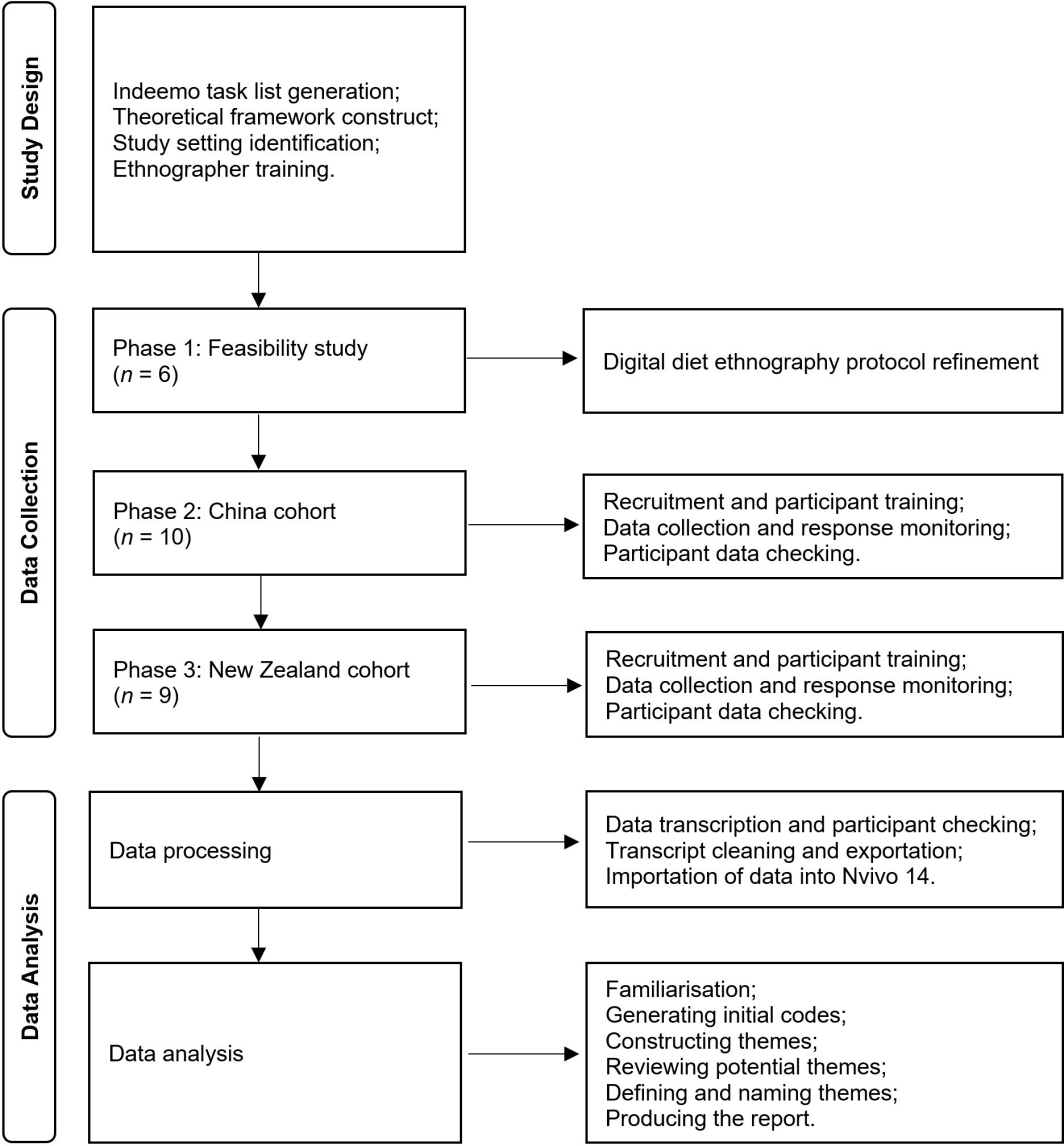
Methods flow chart showing a coherent methodological workflow including study design, ethnography execution, data collection, data processing, and data analysis. Data analysis followed the process published by Terry et al. (42). Figure adapted from Page et al. (96).

The reason for choosing a digital, rather than conventional, diet ethnography was that participants were hard-to-reach via conventional methods in China during COVID-19 pandemic (35). This study was completed in three phases, as summarized in Figure 1. Phase 1 included a feasibility study in China where university students reported eating occasions via a social networking mobile application. Phase 2 included the China cohort and phase 3 included the New Zealand cohort of the main digital diet ethnography, respectively. In this section, we provide necessary details of the theoretical framework; role of the ethnographers; the food environment setting; recruitment, data collection, and data analysis methods. For full details of the methods, please refer to Supplementary file 1. We followed the Consolidated Criteria for Reporting Qualitative Research (COREQ) guidelines to maintain thorough and transparent reporting throughout the study (36) (Supplementary file 2).

### 2.2 Theoretical framework

In our digital diet ethnography, a theoretical framework was chosen only to inspire our inductive grounded theory analysis. In the New Zealand cohort, Bandura's Social Cognitive Theory (SCT) was integrated with Bronfenbrenner's Ecological Model to construct a composite theoretical framework, tailored to the university student population (37). In the China cohort, Contento's (38) theoretical framework was employed because both intrapersonal and environmental factors were considered (23).

### 2.3 Research team reflexivity

Reflexivity acknowledges that researchers' backgrounds, experiences, and assumptions inevitably shape data collection, interpretation, and analysis in ethnographic work. The New Zealand cohort was conducted by a male postgraduate student at the University of Auckland as part of his master's thesis. His own nutrition literacy, recent university student identity, and shared experiential context with participants influenced which themes he perceived as salient and how he interpreted participants' rationales for food choices. Although he produced detailed pre- and post-data-collection positionality statements that explicitly addressed his personal and intellectual goals, social identity, unconscious biases, and the implications of a male researcher studying predominantly female participants' eating behaviors, he did not maintain an ongoing reflexive journal during data collection and analysis, limiting the ability to fully trace potential researcher effects in real time.

The China cohort was conducted by a different male postgraduate researcher at the University of Auckland as part of his Ph.D. thesis. Having lived in China for the first 20 years of his life, originating from the same city (Shijiazhuang) as the study campus, and having previously completed a comprehensive food environment audit on that exact campus (29), he possessed deep contextual familiarity with local food culture, dietary practices, and campus-specific constraints, enabling particularly accurate and nuanced interpretation of participants' choices and rationales. His subsequent 10 years of tertiary education in English-speaking countries provided a strong command of English-language dietary behavior theories, allowing seamless translation and theoretical mapping of Chinese-language themes without loss of cultural nuance. Being a university student himself at the time of data collection further reduced social distance and the risk of misinterpretation of student lifestyle factors. Taken together, the researchers' insider student status, cultural knowledge, and prior campus-specific experience strengthened contextual credibility and thematic resonance across both cohorts, while explicit positionality reflections helped mitigate potential biases stemming from gender, personal eating habits, or theoretical preconceptions.

### 2.4 Study setting

The New Zealand cohort was conducted at the University of Auckland, with most students falling between the ages of 21 and 23 (39). Food service was provided by privately-owned food vendors. The campuses were in urban areas in Auckland where external food vendors surrounding the campus were readily accessible. The China cohort was conducted in two urban campuses of Hebei Medical University (HBMU), located in Shijiazhuang City, China. Most students came from middle- to low-income areas. Food service was provided by university-owned, government-subsidized food outlets. All students were boarding mandatorily. In both cohorts, a convenient sample of participants was recruited from the general student population.

### 2.5 Ethical considerations

This study followed standard ethical procedures for digital ethnography (40), with approvals obtained from the University of Auckland Human Participants Ethics Committee (Reference UAHPEC23986, 4 April 2022, for three years) for the New Zealand cohort and the Research Ethics Committee of HBMU (Approval 202300037) for the China cohort. All participants received detailed Participant Information Sheets and signed informed consent forms that outlined the study aims, procedures, potential risks and benefits, voluntary nature of participation, confidentiality measures, de-identification processes, and the right to withdraw at any time. To protect participant welfare, the researchers avoided exploiting any information that could harm self-esteem, emotions, or well-being; maintained awareness of personal and interpersonal contexts; and worked under qualified supervision throughout. Data were collected via the secure, password-protected Indeemo platform, accessible only by the researcher(s); all identifiable information was de-identified prior to analysis, storage, or presentation. Ethnography data were disclosed publicly only with participant permission and full anonymisation. All data were stored on password-protected university servers, with Indeemo data automatically deleted after project completion.

### 2.6 Feasibility study

The China cohort ethnographer pilot-tested the feasibility of digital diet ethnography among 6 students at HBMU between 17 and 23 Jun 2021. Participants provided photos, videos, and text of seven days' dietary intake. Findings from the feasibility study were used to improve the ethnographers' skills of approaching participants and managing their responses.

### 2.7 Recruitment

Participants for the New Zealand cohort were recruited by convenience sampling via University of Auckland social media channels, emailed enquiries to the student researcher, and campus flyers. Eligibility required being a current University of Auckland student (≥18 years), mobile-device literate, willing to record eating occasions, and not enrolled in nutrition degrees. Interested students received a participant information sheet, consent form and background questionnaire, followed by remote onboarding (Indeemo account setup, task instructions, and a tutorial video) and a Slack workspace for ongoing communication and researcher contact. In total, 10 students consented, 1 withdrew, leaving 9 completers; participants received a NZD 100 supermarket voucher. Because recruitment relied on self-selection and digital responsiveness, the New Zealand sample is likely biased toward students who are digitally comfortable, time-available, and willing to be observed. These traits may correlate with higher food autonomy or confidence in sharing meals publicly and may therefore shape both participation and reporting behaviors. In comparative interpretation we therefore treat New Zealand findings as reflective of engaged, technology-willing students rather than the entire campus population, and we interpret cross-cohort contrasts with caution given these selection and engagement differences.

China cohort recruitment used convenience sampling via posters placed at entry points to the three main university canteens. Recruitment quotas were apportioned across canteens and posters remained in place until the target number of expressions of interest was reached (36 expressions overall), from which 10 participants were randomly selected to maximize perceived fairness. Eligibility mirrored the NZ cohort: full-time students, ≥18 years, digital device literate; nutrition majors and those with eating disorders or metabolic disease were excluded (41). Participants received two structured remote training sessions via Tencent Meeting with ethnographer modeling of responses to normalize recording and were supported with WeChat group communication for the entire duration of the ethnography. Incentives were CNY 1,200 paid upon satisfactory completion. The on-site poster strategy and later random selection reduced some self-selection bias but likely attracted students who habitually dine on campus and those available during recruitment windows. The relatively larger end-payment may have influenced adherence and reporting intensity differently than the New Zealand voucher. Furthermore, mandatory boarding and subsidized, university-operated canteen systems mean that China participants' day-to-day food exposure is structurally distinct from the New Zealand cohort, which both shaped observed behaviors and reduces direct comparability. We therefore frame cross-cohort contrasts as context-sensitive: differences may arise from genuine cultural and institutional factors but also from recruitment modalities, incentive structures, and participant selection processes.

### 2.8 Data collection

#### 2.8.1 Duration

Both cohorts selected a 3-month period because the usual duration of diet ethnography were generally 3 months, with some situated at 6–18 months (17). In the New Zealand cohort, data collection began on 18 Jul 2022 and continued for 3 months, finishing on 01 Nov 2022. In the China cohort, data was collected for 90 days from 08 May 2022 to 05 Aug 2022, inclusively. The China cohort data collection was divided into two tiers because HBMU commenced summer holidays since 08 Jun 2022. Tier 1 included responses from 08 May to 13 or 14 Jun 2022 where participants were boarding on campus and were exposed to the university food environment. Hence, the dietary behavior dataset for tier 1 was named “on-campus”. Two participants returned home on 13 Jun 2022 and eight participants returned home on 14 Jun 2022. Tier 2 included responses from 14 or 15 Jun 2022 to 05 Aug 2022 where participants were exposed to a wide variety of food environments, mainly home but also workplaces (hospitals), tourist cities/sites, and in a different university. Hence, the dietary behavior dataset for tier 2 was named “at-home”.

#### 2.8.2 Type of data collected

In both cohorts, data was collected using Indeemo, a digital ethnography platform. Participants were required to respond to four tasks on each calendar day: one each for breakfast, lunch, dinner, and snacks. When creating a response, participants could decide to take a photo, record a video, write a text description, or a combination of all three forms. In addition, the New Zealand ethnographer conducted interviews retrospectively to address unanswered questions.

#### 2.8.3 Monitoring participant responses

Keeping track of what the participant had been doing informed the ethnographer of any immediate actions that needed to be taken. Both ethnographers allocated a regular period, usually 09:30–10:30 a.m., to examined participant responses every day. The ethnographers prompted the participants for any issues with their responses.

### 2.9 Data analysis

Data were analyzed using a hybrid inductive–abductive approach that drew on grounded-theory principles and the six-phase thematic procedures described by Terry et al. (42). In practice, inductive codes were generated from participants' transcripts and media (videos, photos, captions), and these initial codes were iteratively compared and, where appropriate, mapped to sensitizing concepts from corresponding theoretical frameworks in each cohort. Deductive codes from the frameworks were therefore used as helpful analytic guides, while inductive codes were retained whenever participant material did not fit existing constructs or represented novel meanings. The primary ethnographer completed initial coding for each cohort; coding lists were discussed in regular meetings with a supervising researcher, and unresolved disagreements were adjudicated by a second senior investigator. Although the two cohorts used different theoretical starting points, both ethnographers adhered to the same qualitative content analysis procedures, maintained harmonized coding practices. We monitored conceptual saturation by coding meal entries in chronological order and tracking cumulative unique codes: saturation was pragmatically defined as fewer than 1 new code per 10 successive meal entries combined with the absence of new substantive themes across two consecutive weeks. Using this criterion, initial coding reached saturation approximately by day 13 (range 11–17) for on-campus diets for both cohorts, and day 15 (range 11–19) for at-home diets in China cohort. Nevertheless, all data were coded in full to allow exploratory theme development. Automated Indeemo transcripts were manually verified where ethnographers reviewed every original video and audio entry, corrected transcription errors, and logged corrections in NVivo to ensure data fidelity prior to analysis.

### 2.10 The Hawthorne effect

Ethnographers experience significant barrier to draw valid conclusions when participants divert from natural behaviors, known as the Hawthorne effect (43, 44). In this study, we implemented mitigation strategies including training, prolonged data collection, and participant-as-observer posting, which may have reduced reactivity over time. Nevertheless, some Hawthorne-type reactivity remains possible. Participants were encouraged to respond with a video recording of their eating occasion instead of simple text. Two training sessions were provided by the China cohort ethnographer to familiarize participants with study expectations and Indeemo tasks. Both cohorts collected data over a long period of time, allowing rapport and trust to be established between the ethnographer and the participants (45, 46).

Among all practices, perhaps those minimizing the participants' feeling of being observed were the most effective (47–49). For example, the China cohort ethnographer created a group chat via a mobile social networking application, WeChat (Tencent, Shenzhen, China), where all participants had access to. The ethnographer published every eating occasion as his own responses, every day for 90 consecutive days, exactly as the participants were expected to do. When the participants became the “observer”, their responses exhibited more natural settings.

## 3 Results

### 3.1 Overview

Participant adherence during the digital diet ethnography was adequate and provided satisfactory video, graphic, and text data. A total of nine students, all females aged between 19 and 29 years (mean age 22 years), completed the New Zealand cohort digital diet ethnography. A total of 10 participants (six females) with an average age between 20 and 23 years (mean age 21 years) completed the China cohort. This section summarized participant behaviors during the digital ethnography and the results of qualitative content analysis. Findings from the New Zealand cohort were presented first, highlighting the four themes and how their sub-themes related to university student dietary behaviors. Then, three major themes and associating codes from the China cohort were presented, including on-campus and at-home datasets. A comparative synthesis between the two cohorts was then presented. Remarkable extracts from participant responses were included where appropriate.

### 3.2 New Zealand cohort

Nine University of Auckland students participated in the New Zealand cohort of this digital diet ethnography (all females; age range 19–29 years), producing a three-month dataset, describing everyday food practices. Thematic analysis of the New Zealand arm identified four principal themes: time is of the essence, cash is king, the on-off campus conundrum, and miscellaneous influences. Each principal theme contained several interrelated subthemes that capture how students made food choices within their academic and social lives, as shown in Table 1.

**Table 1:** New Zealand cohort major themes and associated sub-themes.

**Time is of the essence**	**Cash is king**	**The on-off campus conundrum**	**Miscellaneous influences**
The importance of convenience	Strategies to ameliorate food cost	Studying on-campus vs. off-campus	Food and nutrition knowledge
Healthy eating	Food and flat mates	The on-campus food supply	Family
Meal prepping	Friends and spending		Wastefulness
University commitments	Healthy eating		Location of residence
Stress, fatigue and tiredness	Food cost while living at home		Halls of residence
Times of heavy academic load			COVID-19
Snacking and studying			Social media

#### 3.2.1 Time is of the essence

Across the theme time is of the essence, participants repeatedly emphasized the primacy of convenience in shaping moment-to-moment food choices. During periods of heavy academic workload, short breaks between classes or clinical placements, and evening study sessions, students reported defaulting to quick, ready-to-eat options or skipping structured meals entirely. These temporal constraints interacted with physiological states, including hunger, fatigue and stress, to encourage grab-and-go purchases, snacking while studying, and reliance on pre-packaged foods that could be consumed between commitments. Participants also described strategies intended to manage time pressures, such as meal prepping on less busy days, but noted that such strategies were difficult to sustain during peak academic weeks.

#### 3.2.2 Cash is king

The theme of cash is king captured the pervasive role of financial considerations. Students described a constant cost-benefit calculus in which price, portion size and perceived value determined whether they purchased food on campus or sought alternatives off campus. Many respondents used cost-saving strategies such as sharing takeaways with flat mates, choosing cheaper vendor options, or timing purchases around discounts. Meanwhile, some reported that limited budgets constrained their ability to prioritize healthier items. At home, different economic dynamics applied. When living with family, some students noted lower out-of-pocket food costs but also different priorities around meal composition and household budgeting. This emphasis on affordability and the tactical responses to it were recurring components of the New Zealand cohort.

#### 3.2.3 The on–off campus conundrum

The on–off campus conundrum describes how physical food environments and institutional arrangements shaped food choices. On campus, students encountered a mix of privately operated vendors and campus concessions. Participants commented on the convenience and rapid service these outlets offered but also on limited healthy options, high prices for some items, and frequent sensory cues promoting energy-dense choices. Off campus, supermarkets and local eateries often provided greater variety and the potential for cheaper, healthier choices. However, accessing these alternatives required time and transport. Consequently, students negotiated trade-offs between convenience and quality: if time was short, they bought on campus despite perceived lower healthfulness, but when schedules permitted, they opted off campus for greater diversity or value. Students proposed longer opening hours, clearer nutrition labeling, and more affordable healthy meals on campus to reduce this tension.

#### 3.2.4 Miscellaneous influences

Miscellaneous influences encompassed a range of intrapersonal, social and societal factors that moderated food choices. Nutrition knowledge and cooking skill influenced some participants' capacity to prepare balanced meals, whereas family background and flatmate practices shaped habitual preferences and food routines. Waste concerns, residential location, the impacts of COVID-19 on living arrangements and meal patterns, and the role of social media in introducing new foods or dieting narratives were also evident in the dataset. Social media, in particular, functioned both as an enabler, encouraging group outings to new restaurants and sharing recipes, and as a source of potentially misleading diet information. Participants discussed both the inspiration and the pressures they encountered online.

### 3.3 China cohort

A total of 10 participants (six females) with an average age of 22 years (range 21–23 years) completed the China cohort, producing a three-month dataset, describing everyday food practices. Thematic analysis revealed three major themes that structured students' dietary behaviors: social and environmental determinants, food-related determinants, and intrapersonal determinants, as shown in Table 2. These themes manifested differently across on-campus and at-home settings. Below we described each domain and its major subthemes, using illustrative extracts where appropriate.

**Table 2:** Summary of China cohort dietary behavior determinants with comparison between on campus and at home datasets, illustrated by direct short quotes.

**Themes**	**Major subthemes**	**Minor subthemes**		**Short quotes**
		**On campus**	**At home**	
Social and environmental	Price, food environment, interpersonal factors, university lifestyle, social conditioning, home lifestyle, social and cultural environment	Accessibility, convenience, availability, quality, partner, schoolmates, social and cultural environment, cultural practices, social setting, social structure and policies, social conditioning, meal-skipping	Family members, availability, accessibility, quality, convenience, food parenting practices, regional diet norms, meal routines, cultural practices, university lifestyle, economic environment	“*I got up quite early, but I just felt too lazy … I skipped breakfast*” “…* because this was convenient to carry and my roommates liked these noodles … I bought them*” “… *when I'm at home, I eat whatever my parents cook*” “*Tonight, mom cooked loofa egg soup. The loofa was grown in our own backyard, it's fresh and healthy*”
Food-related	Food preferences, desirable foods, pleasure, satiety and hunger, physiological experiences	Energy-dense foods, taste, texture, physiological experience, aversions, climate, ease discomfort, exposure to new foods, positive feelings when eating, familiarity	Hungry, not hungry, thirst, taste, texture, urge to eat, liked foods, reluctance, familiarity, not eaten for a while, aversions, weather	“*This is what I'm having for dinner, rice roll, it's seriously delicious*” “…* I have not had dumplings for several days. So, I went to buy dumplings for lunch*” “*I've been eating roujiamo at this retailer since when I was a child … I'm dying to have one*”
Intrapersonal	Nutrition knowledge and skills, motivating factors	Nutrient intake, portion size estimation, read nutrition labels, body image management, dietary pattern, food function, healthiness, meal planning, mood	Nutrient intake, portion size estimation, planning, food function, weight management	“*I had a creperie and one cup of black rice porridge … shredded cabbage and pork loin … I call it the balanced diet*” “*Dad said he wanted to cook sugar tomatoes … he didn't know how to make that dish, needless to say my cooking skills. So, we had to give up that plan*”

#### 3.3.1 On-campus findings

On campus, social and environmental determinants were the most prominent influence on students' eating patterns. Students at HBMU are automatically assigned to 8-person dormitories and typically share academic timetables with their roommates. This proximity fostered strong interpersonal influence over meal selection and routine eating practices. Good value-for-money and convenience were repeatedly invoked as drivers. Participants described comparing portion size and taste against price, avoiding long queues, and favoring foods that were quick to consume or easy to carry. One participant captured the role of partners in on-campus feeding decisions: “*Today I had stewed beef with tomatoes served with rice, my partner bought this for me, so I didn't know in advance*” (**Extract 1, F3**). Social occasions and shared orders similarly lowered barriers to consuming less healthy or more expensive foods, as another participant recounted: “*We ordered roast chicken and beef kebab; my girlfriend ordered two jumbo size 1 L bubble tea and brought her biscuits for sharing among us. Let's eat!*” (**Extract 2, M4**). University scheduling and academic stress also shaped eating. Tight timetables led some students to skip meals, while exam-related stress increased reliance on convenient, energy-dense foods.

Within food-related determinants, taste and sensory appeal such as flavor, texture, and familiarity strongly guided choices. Visual cues and urges to eat could frequently override nutrition knowledge. For example, participants described seeking familiar, flavourful options and sometimes growing tired of repetitive canteen menus. Physiological states such as hunger and satiety also influenced portion size and selection. Many students reported preferring foods that provided immediate satisfaction and comfort during long study days.

Intrapersonal determinants were present but less dominant in the on-campus dataset. Nutrition knowledge, body-image concerns, and meal planning appeared intermittently. Some participants deliberately limited carbohydrate intakes for weight management, while others described routine snack behaviors or variable appetite linked to mood. Overall, individual agency was constrained by the broader social and institutional environment, limiting students' ability to act on nutrition knowledge.

#### 3.3.2 At-home findings

When participants described eating at home, social and environmental determinants again predominated, but with a different social actor: parents. Family food practices, meal routines, and parental decisions strongly determined what students ate at home. Most participants reported generally eating whatever their parents prepared. One participant illustrated this dynamic: “*Breakfast is ready now. Mom asked me if I'd like to have stir fried onion with eggs, I said no. So, she cooked stir fried onions for me. But it's actually the onions that I didn't want to eat, not the eggs*” (**Extract 3, F5**). The home food environment featured readily accessible snacks, home-grown vegetables, and different patterns of food availability. While reducing some access barriers seen on campus, this simultaneously limited autonomy when meal composition and timing were controlled by parents.

In the food-related domain at home, sensory preferences and familiarity again predominated. Participants described pleasure in home dishes and an inclination to eat foods perceived as delicious or culturally familiar. One participant listed a varied meal including tofu soup and vegetable salad, concluding that the spread was “*seriously delicious*” (**Extract 4, F4**). The at-home context also enabled some experimentation with recipes and small acts of food preparation, although opportunities for autonomous cooking varied by family circumstances and respondents' skills.

Intrapersonal determinants at home included nutrition knowledge, food skills, and culturally embedded beliefs about food function. Several participants articulated traditional perceptions of foods' health effects and family practices often transmitted these beliefs. For example, describing millet porridge as “*nourishing my stomach*” (**Extract 5, F5**). Practical cooking skills varied where some students reported limited ability to prepare wished-for dishes, which constrained attempts to influence household menus.

### 3.4 Cross-cohort comparison

Across both cohorts, three broad patterns consistently influenced student food choices: structural constraints (time and food availability), economic pressures, and moment-to-moment sensory and physiological drivers. In New Zealand, these pressures most often translated into trade-offs between convenience and quality. Students with limited time tended to choose privately sold, ready-to-eat options on campus and only traveled off campus when schedules and budgets allowed. Cost considerations operated as a constant cost-benefit calculus, prompting sharing strategies, discount-seeking and substitution toward cheaper, energy-dense items. In China, the same categories of determinants were evident, but their practical expression followed different institutional logics. Campus canteens and mandatory dormitory living concentrated social influence and shaped routine meal patterns, while parents exercised primary control over food at home. In both settings, sensory appeal, hunger, and comfort repeatedly overrode abstract nutrition knowledge, but these immediate drivers played out against distinct backdrops of vendor models, meal provisioning and daily schedules.

Despite these shared determinants, the mechanisms generating them diverged in ways that have clear cultural and institutional roots. New Zealand students generally reported greater individual autonomy and flexibility. They negotiated between on-campus convenience and off-campus options depending on time and money whereas Chinese students experienced more constrained autonomy arising from collective living arrangements, government-subsidized canteen structures, and parental decision-making at home. Consequently, interpersonal influence was a central force in both cohorts but with different primary actors: peers/roommates in China on campus; parents in China at home; more individualized peer influence and autonomous decision-making in New Zealand. These contrasts suggest that although the categories of determinants are similar across contexts, the pathways by which they shape behavior are context dependent.

Taken together, these convergences and divergences form the empirical basis for the comparative interpretation that follows. The Discussion therefore examines how cultural norms, institutional food systems and economic constraints interact to produce the observed patterns of diet autonomy and choice in each setting, and it considers implications for university food policy and interventions that are sensitive to these contextual differences.

## 4 Discussion

### 4.1 Overview

This digital diet ethnography provides the first in-depth qualitative comparison of dietary behavior determinants among university students in two markedly different food-environment contexts: a decentralized, commercialized New Zealand campus and a centralized, government-subsidized Chinese medical university. Despite structural differences, both cohorts revealed similar core dietary behavior determinants: time scarcity, financial constraint, convenience-seeking, taste/familiarity preferences, and social influence. These drivers often operate as powerful barriers to healthy eating while interacting dynamically with the respective food environments to produce context-specific patterns (50–52). New Zealand students exhibited high food autonomy but frequent reliance on cheap, convenient, energy-dense options, while Chinese students displayed lower day-to-day autonomy yet greater exposure to interpersonal and cultural norms that shaped choices. The richness of the 90-day dataset (approximately 2,700 eating occasions per cohort) and the ethnographic depth enabled identification of potentially modifiable factors, including availability, affordability, social norms, convenience infrastructure, and sensory appeal. Consistency of such factors across cultures reinforces the utility of ecological models for understanding university student dietary behavior (38, 51). The findings extend previous qualitative work in Western (50, 53, 54) and Asian settings (55, 56) by demonstrating how macro-level food-system differences (profit-driven vs. welfare-oriented) translate into micro-level lived experiences among university students (23).

### 4.2 Time scarcity and convenience as universal drivers

Time emerged as the dominant constraint in both cohorts. New Zealand participants repeatedly framed food choice as a trade-off against academic workloads, with convenience trumping health during lectures, assignments, and exam periods. Chinese students similarly prioritized speed and proximity but within the structural reality of fixed canteen hours, long queues, and rigid class schedules. In this setting, meal-skipping or instant noodles were common coping strategies (57). The key difference lay in recovery opportunities: New Zealand students could access 24/7 off-campus options, whereas Chinese students on campus were captive to operating hours and had fewer alternatives when canteens closed. When Chinese participants returned home for summer break, time pressure eased and dietary variety increased markedly. Such environmental shift highlights how rigidly structured campus routines, rather than inherent student laziness, could drive convenience-seeking behavior (23, 52).

### 4.3 Financial pressures: different structures, same outcome

Cost was described as “cash is king” in New Zealand and an ever-present consideration in China despite government subsidies. High food autonomy among New Zealand students allowed sophisticated cost-minimization strategies, yet healthy foods were repeatedly labeled “too expensive,” leading to deliberate trade-offs (53, 54). Chinese students benefited from canteen prices substantially lower than off-campus equivalents yet still perceived healthy options as relatively costly within their limited budgets, suggesting that subsidies reduced absolute prices but did not eliminate relative cost barriers (29). Both cohorts therefore converged on energy-dense, nutrient-poor choices as rational economic responses to perceived expensiveness (58), confirming that affordability remains a critical determinant even in subsidized systems when healthier items carry premium pricing or preparation time costs.

### 4.4 Social influences: individualistic vs. collectivistic expressions

Social factors operated differently yet with comparable strength. New Zealand participants were heavily influenced by flatmates, friends, and partners, but decisions remained ultimately individual. Chinese students exhibited stronger collectivistic patterning: roommates synchronized meals, shared food purchases, and exerted normative pressure. Furthermore, family control was near-absolute when students returned home. This reflects broader cultural orientations, i.e. individualism in New Zealand vs. collectivism in China. However, the behavioral outcome was similar: social context frequently overrode personal health intentions (59). The China cohort provide particularly strong evidence of social conditioning as a modifiable determinant, with peer purchasing behavior and family food parenting practices exerting powerful effects that persisted beyond the campus environment (60).

### 4.5 Food-related determinants: taste, familiarity, and sensory pleasure

Both cohorts prioritized taste, texture, and familiarity over nutrition messaging. New Zealand students sought “comfort” and “reward” foods during stress, often articulating guilt but prioritizing immediate sensory pleasure. Chinese participants displayed even stronger attachment to familiar flavors and regional dishes, with nostalgia and childhood memories frequently cited. Exposure to new foods was limited in both settings by risk aversion and habitual choice, but the Chinese cohort showed greater willingness to try novel items when socially facilitated or during holidays. These findings support Köster's psychological perspective on food choice diversity (61) and demonstrate that sensory and emotional functions of food consistently outweigh cognitive health knowledge across cultures (62).

### 4.6 Intrapersonal factors and the autonomy: constraint paradox

New Zealand students possessed high behavioral control yet frequently chose not to exercise it in favor of health, citing fatigue, stress, and competing priorities. Chinese students on campus had lower control due to fixed menus and limited cooking facilities but showed surprisingly high acceptance of this constraint, framing it as normal rather than problematic. When autonomy increased at home, healthier patterns emerged for some but not all, highlighting that autonomy alone is insufficient without supportive environmental conditions (63). Nutrition knowledge was present but rarely actioned in either cohort, which was consistent with global evidence that knowledge-behavior gaps persist in young adults (64–66).

### 4.7 Structural food environment as the ultimate mediator

The most profound differences arose from institutional structure rather than culture *per se*. New Zealand's commercialized, fragmented foodscape produced high variety but pervasive unhealthy default options and price signaling that penalized health (10, 23). China's centralized, subsidized system delivered superior affordability and reduced food insecurity but at the cost of variety, flexibility, and nutritional quality, creating a different but equally potent set of constraints (29). Both systems need improvement to make healthy choices the easy, or default, choice, confirming that individual-level interventions alone are inadequate when structural determinants dominate decision-making contexts (67, 68).

Overall, the cross-cohort comparison demonstrates that university students' dietary patterns are primarily shaped by pragmatic adaptations to common contextual constraints rather than individual shortcomings. These constraints exert remarkably consistent influences on eating behavior despite different institutional and structural approaches to food provision in the New Zealand and Chinese university settings. Interventions must therefore target the shared modifiable determinants, including time-compatible healthy options, genuine affordability of nutrient-dense foods, social norm change, and sensory appeal, while exploiting each food environment system's strengths to shift “default” dietary choices toward healthier options (69, 70).

### 4.8 Strengths and limitations

#### 4.8.1 Strengths

As the first digital diet ethnography to investigate diet behavior determinants conducted in New Zealand and Chinese university food environment settings, this study has several unique strengths. In this research topic, existing literature primarily used self-designed, close-end questionnaires to collect cross-sectional data on food choices and dietary behavior determinants (18, 71–73). As shown in our feasibility study, participants experience significant barriers to organize and express their perceptions, attitudes, and thoughts toward their dietary behaviors. Digital diet ethnography elicited in-depth participant responses in qualitative manners, including audiovisual, graphic, and textual data in natural eating occasions (74).

In terms of approaching participants, individual interviews have been found to be more powerful in exploring a wider range of themes when compared to a focus group in qualitative research (75). In this study, the nature of interaction between the ethnographer and each participant closely resembles an interview but adds further dimensions that conventional free-listing interviews lack (76). This approach aligned well with our research goal of eliciting both manifest and latent dietary behavior determinants (77). Moreover, the China cohort ethnographer immersed in the study by completing all tasks and posting the responses publicly among all participants. Such actions alleviated participants' perceptions of “being observed” in an ethnographic study and promoted authentic responses in their natural eating occasions.

#### 4.8.2 Limitations

Despite our strengths, the most salient limitation was the exclusion of nutrient intake assessment during data collection. Participants described their eating occasion, reasons for food choices, and what foods were eaten. However, they were neither trained for portion size estimation nor asked to weight the foods they ate, which meant that the researcher could not make objective evaluation of the healthiness of participants' diets. Future research could incorporate these measures to further understand the association between dietary determinants and dietary outcomes (78). Studies in diverse settings have identified additional themes of interest not addressed in our study. For example, none of our participants reported smoking or alcohol consumption as a determinant (57, 79). Furthermore, participants rarely reported on health or nutritional concerns while making dietary decisions despite potentially higher nutrition literacy (80). Future research could develop and validate a tool for measuring health literacy and health status among Chinese university students, addressing their age- and culture-specific features.

Convenience sampling may have introduced selection bias, as students who volunteered may differ from the broader student population in motivation, digital literacy, or interest in nutrition. In China, participants willing to engage in long-term digital self-reporting may represent students with stronger peer networks or greater conscientiousness, potentially amplifying interpersonal influences observed in this study. In New Zealand, participants comfortable with video or photo reporting may be those with higher food autonomy or greater confidence in documenting their meals. These characteristics may limit generalisability; however, such limitations are common in ethnographic and exploratory qualitative research, which prioritizes depth of insight over representativeness. The modest sample sizes and differences in methodological frameworks between the two countries also limit the extent to which causal or structural comparisons can be drawn. Nonetheless, the cross-cultural contrasts provide valuable groundwork for future mixed-methods and longitudinal studies.

Despite many countermeasures to minimize the risk of introducing observer bias, findings from this study might still not be generalisable (81). With future ethnography in university settings, a synthesis of findings warrants a theoretical framework tailored for dietary behavior determinants among university students (82). Compared with conventional ethnography, digital diet ethnography also risk losing potential non-verbal cues, which could sometimes reveal key themes (83, 84). Misinterpretation could also occur with digital communication when the researcher attempted to interpret self-recorded videos from participants. Using wearable devices with video and audio recording capabilities in future research could mitigate this issue. Furthermore, our sample size was standard for ethnographic studies, ranging from 6 to 20, but still relatively small (76, 85, 86). The participant demographic homogeneity partly limited the generalisability of our findings among global university students.

### 4.9 Implications

These findings have direct implications for university food policy and student health promotion in both New Zealand and China. Universities could strengthen campus food environments by prioritizing the accessibility, affordability, and appeal of nutritious options through procurement policies aligned with public health nutrition guidelines and sustainability principles, thereby reducing student reliance on low-cost, energy-dense foods (87). New Zealand institutions would benefit from stronger policy coordination and regulatory oversight to ensure vendors offer nutritious, culturally responsive choices (88). Chinese universities can implement system-level reforms to enhance menu diversity and nutritional quality, especially with centralized food-service systems (89). Complementary interventions should foster greater student food autonomy: in New Zealand through improved nutrition education, cost-effective meal-preparation support, and food-insecurity alleviation; in China via family engagement, cooking-skill development, and culturally tailored guidance. Finally, both countries should integrate environmental sustainability into food pricing, messaging, and offerings while ensuring such initiatives remain economically accessible to students.

The findings of this ethnography offer methodological implications for future qualitative and quantitative studies of university student dietary behavior. To minimize bias and enhance data quality, future digital or face-to-face diet ethnographies should prioritize prolonged pre-recruitment engagement to build rapport and incorporate barrier-free data collection (90). Researcher training in task design, culturally sensitive communication, and empathy remains essential to mitigate observer effects in context-specific settings (91). Demographics, socioeconomic status, and hometown should be routinely collected as predictors for subsequent quantitative analyses (53). Digital technologies, including mobile ethnography apps and wearables, substantially reduce the observer effect while enabling simultaneous capture of quantifiable intake metrics and rich qualitative rationales, despite ongoing ethical discussions (45, 92–94). Potential next quantitative steps are therefore to (1) assess diet quality and health status using validated instruments such as Healthy Eating Index, (2) implement or leverage digitized campus food-service systems for precise intake recording (95), and (3) conduct controlled intervention trials evaluating food environment modifications on student dietary outcomes.

## 5 Conclusions

This cross-cohort digital diet ethnography demonstrates that university students' dietary behaviors are shaped predominantly by structural and social contexts, notably time pressure, cost considerations, convenience, sensory appeal and interpersonal influence, rather than simply by individual lack of knowledge. While New Zealand students had greater day-to-day autonomy, they frequently defaulted to inexpensive, convenient, energy-dense foods. Chinese students experienced more constrained autonomy on campus but stronger peer and family influences that shaped choices both on campus and at home. Our three-month, multimodal approach generated contextually grounded insights with methodological rigor strengthened through harmonized coding procedures, reflexivity reporting, and manual verification of AI transcriptions. Nevertheless, findings are exploratory: convenience sampling, cohort differences in recruitment and incentives, modest sample sizes, and the absence of quantified nutrient intake limit generalisability. Policy and practice responses should therefore be context sensitive, for example, menu diversification and reformulation within subsidized systems, and procurement, pricing and vendor-contract standards within marketized systems. Promoting time-compatible, affordable, and palatable healthy options and leveraging social networks to shift norms could potentially be effective. Future mixed-methods and intervention studies are needed to quantify effects and evaluate structural changes that make healthier choices the default on university campuses.

## Data Availability

The raw data supporting the conclusions of this article will be made available by the authors, without undue reservation.
