# Supplementary material for: How do they eat: a digital diet ethnography of dietary behavior determinants among New Zealand and Chinese university students
Source: Front Nutr. 2026 Jan 12;12:1729437. doi: 10.3389/fnut.2025.1729437 (PMC12832373; doi:10.3389/fnut.2025.1729437)
Supplement: Supplementary file 1 [file Table_1.docx]

Supplementary Material

# Additional file 1: digital diet ethnography protocol

## Overview

This digital diet ethnography project was conducted in three phases. Phase 1 included a feasibility study in China where university students reported eating occasions via a social networking mobile application. A standard operating protocol for conducting digital diet ethnography among university students was written after phase 1 was completed. In compliance with the standard operating protocol, phase 2 included the China cohort and phase 3 included the New Zealand cohort, which began two months after phase 2 commenced. However, the actual digital diet ethnography practices differed slightly between New Zealand and China due to many reasons, which were articulated in appropriate parts of the following section.

To date, neither conventional nor digital diet ethnography studies conducted within Chinese university setting were reported. The paramount reason for choosing a digital, rather than conventional, diet ethnography was that COVID-19 lockdown policies were still active in China during the period when this project was conducted, and participants were hard-to-reach via conventional methods [1]. University students exhibit high literacy for using digital devices, which facilitated participant engagement via digital platforms, mobile applications, and response monitoring [2]. Digital ethnography is capable of capturing the situation surrounding student behaviour, allowing the eating occasion to be comprehensively recorded [3]. In addition, compared to conventional ethnography, integrating digitised tools was found to partly mitigate the observer effect and allow students to experience a more natural research environment [4]. As the first of its kind, this study attempted to establish a systematic strategy of executing digital diet ethnography among this population, such that findings from this and similar digital diet ethnography are based on quality data.

In this section, we first conceptualised the theoretical framework that was used to guide our research design. We defined the role of the researcher in digital diet ethnography. Then, we described the food environment setting that our ethnographic study was conducted, and how potentially competent participants were recruited and selected. Subsequently, data collection methods and participant compliance strategies were reported. Finally, we described how grounded theory and qualitative content analysis was used and adapted as our choice of data analysis.

## Theoretical Framework

We employed grounded theory analysis to explore university student dietary behaviour determinants in New Zealand and China. In grounded theory studies, a preconceived theory is usually absent since the codes and categories were synthesised inductively from the data. However, producing a comprehensive dietary behaviour determinant theory based on qualitative data requires extensive background knowledge and expertise in ethnographic research, especially when the setting is an understudied field [5]. Hence, an existing theoretical framework was necessary in this case to complement inductive coding and to enable abductive reasoning [6].

In the New Zealand cohort, we identified two theoretical frameworks that have been widely used to study dietary behaviours under comparable cultural settings [7-10]. First, Bandura’s Social Cognitive Theory (SCT) suggested that student dietary behaviours are influenced or determined by a complex, reciprocal interplay of three factors: personal cognitive factors, socioenvironmental influences, and behavioural factors [11]. Then, we integrated SCT with Bronfenbrenner’s Ecological Model to construct a composite theoretical framework, tailored to the university student population [12]. The final four integrated levels of dietary behaviour determinants were: individual or intrapersonal influences, social environmental or interpersonal influences, physical environmental or community influences, and macrosystem or societal influences. In the composite framework, we defined the “physical” food environment as physical environmental influences, and the “nonphysical” food environment as intrapersonal, social environmental and societal influences.

In the China cohort, retrieving a tailored theoretical framework for dietary behaviour determinant among Chinese residents was unsuccessful through literature search and expert consultation. We examined several potentially suitable frameworks developed in Western countries. Psychological determinant frameworks were common, but interventions based on these frameworks showed modest effects [13]. Contento’s [14] theoretical framework considered both intrapersonal and environmental factors, and substantially agreed with previous findings on university students’ dietary behaviour determinants [15]. Hence, this framework was used to inform inductive coding by indicating three major themes of dietary behaviour determinants: 1) food preferences/reluctance; 2) personal food choice motivations or intrapersonal norms or practices, and 3) social/environmental influences.

## Researcher Positionality

The way ethnographers collect, perceive, and analyse ethnography data could be shaped by their assumptions, prior experience, and background knowledge [16]. The New Zealand cohort was completed by a male postgraduate student from the University of Auckland as part of his master’s thesis. The nutrition literacy, social identity, and university experience would have likely contributed towards the themes that he deemed to be significant. It is also important to recognise that he had a very limited understanding of ethnography, content analysis, and qualitative research. The China cohort was completed by a male postgraduate student from the University of Auckland as part of his PhD thesis. The China cohort ethnographer has spent over 10 years in English-speaking countries, completing secondary and tertiary education. The dual cultural background enabled him to relate participant responses in Chinese language to English-language dietary behaviour theories. Furthermore, a previous university food environment audit was conducted by the ethnographer in the exact campus that all participants were at [17], which further strengthened the connection between the researcher and the participants’ food environment.

## Study Setting

### The New Zealand cohort

The New Zealand cohort was conducted at The University of Auckland, a public research university located in Auckland, New Zealand. The University of Auckland is the tertiary education provider of 46,000 students, with 8000 students studying internationally and 36,000 students studying locally in New Zealand. A diverse mixture of genders and ethnicities make up the student demographic, with most students falling between the ages of 21 and 23 [18]. The target population was students that attended the University of Auckland at the time of recruitment and throughout the proposed research period. Initially, students chosen to participate in the research were intended to be students that regularly purchased food from university food outlets. However, due to limited expression of interest, participants were chosen from the general student population.

### The China cohort

The China cohort was conducted in Hebei Medical University (HBMU), located in Shijiazhuang City, China. HBMU currently holds approximately 14,000 undergraduate students and 6,000 postgraduate students. It has two urban campuses, namely Zhongshan campus and Jianhua campus. Students in HBMU are automatically assigned to 8-person dormitories upon enrolment. Dormitories were organized to accommodate students who attended the same lectures or laboratory sessions. Hence, roommates share similar daily routines and were likely to influence each other’s dietary behaviours [19]. In this study, we targeted full-time students enrolled at HBMU, including both undergraduate and postgraduate students.

## Ethical considerations

Ethical approval for the New Zealand cohort was obtained from the University of Auckland Human Participants Ethics Committee on 04 Apr 2022 for three years (approval number UAHPEC23986). The China cohort of this digital diet ethnography was approved on 17 Nov 2022 by the Institutional Review Board of The First Hospital of Hebei Medical University for two years (approval number 2023YSD00037). In both arms, participants gave informed consent by signing a consent form prior to participation in the study. Full aims and procedures were disclosed to participants in the informed consent form.

The ethnographers avoided exploiting information from participants in ways that would harm their self-esteem, emotion, or any other aspects. Participant uploaded responses to Indeemo, an online qualitative research platform. Password is required to access participant responses and personal information. The ethnography data was only viewed, analysed, and transported by the two ethnographers only. Text or photos from ethnography data were only disclosed, at the participants’ discretion, at academic conferences or seminars with all personal information de-identified.

## Feasibility Study

Between 17 and 23 Jun 2021, the China cohort ethnographer pilot-tested the feasibility of digital diet ethnography among 6 students at HBMU to inform the methodology of our main study. Participants provided photos, videos, and text of seven days’ dietary intake. At the beginning, the ethnographer gave an example of eating occasion response of “*stating where the food was purchased, where the food was consumed, what was consumed, and how much did the food cost*”. All participants, however, only provided some or all of the above information in subsequent responses. The ethnographer noticed this issue after viewing participant responses by the end of day 1 and verbally encouraged the participants to describe their eating occasions, which unfortunately was ineffective at all. Although the quality of participant data was unsatisfactory, the task completion rate was 100%. The ethnographer interpreted this observation as a result of convenience sampling of approachable, subordinate undergraduate students.

In the formal digital diet ethnography study, the ethnographer increased the frequency of online video communication with the participants. Language and tone of communication was less formal. The China cohort ethnographer posted videos of his own eating occasion in social networking applications so that participants felt like being “in command” of their participation in this study. Findings from the feasibility study further informed multiple aspects of the recruitment and training during the formal digital diet ethnography.

## Recruitment

### The New Zealand cohort

The digital diet ethnography was advertised using University of Auckland social media channels, and advertisement flyers were placed around University of Auckland campuses located within central Auckland. Participants were recruited using convenience sampling where the first students to express interest and meet the eligibility criteria were recruited. To meet the eligibility criteria, students were required to study at the University of Auckland at the time of expressing interest and throughout the proposed research period; be over 18 years of age; live independently with the ability to give their own informed consent; have access to a mobile device that could run the Indeemo application (smartphone with iOS 10 and above or Android 5 and above) and be willing to record eating occasions using the Indeemo application over a period of 3 months in 2022. Students that had studied or were studying a Bachelor of Science in Nutrition or a Bachelor of Science in Food Science were also excluded from the study, as their nutrition literacy was not reflective of the general student population. To avoid power dynamics and conflicts of interest, students within the 2021/2022 Masters of Health Sciences in Human Nutrition and Dietetics cohort, students the ethnographer knew personally, or students who reported to the supervising researcher were also excluded. To incentivise participation, participants received a $100 supermarket voucher at the end of the data collection period. Participants were also offered to enter their contact details (name, email and address) for a chance to win a Lenovo M7 Android Tablet. Once a student had expressed interest in participating in the research, they were emailed a participant information sheet, which included a project outline, inclusion criteria, details of participation, potential risks and discomforts, benefits, confidentiality, data storage and publishing of results; a consent form; and a participant information questionnaire to provide background information about the participant. The digital diet ethnography aimed for 10 participants. Of the ten initial included participants, one withdrew from the study, and another was lost to follow up. Because the first participant withdrew early in the data collection period, another student that had expressed interest was randomly selected as a replacement, leading to nine students fully participating in the study (n=9).

### The China cohort

We employed convenience sampling where recruitment information was publicly displayed at the university through a recruitment poster. Participants expressed their interest in taking part in our study by scanning a QR code on the poster to contact the ethnographer via mobile social networking application WeChat (Tencent, Shenzhen, China). We recruited participants who frequently dine on campus by placing the poster at the entrance of three university canteens on two campuses. The target number of participants was 10, and an expression of interest quota of 12 at each canteen was set to allow students of diverse canteen or food preferences to be recruited. At each of the three canteen, recruitment poster was removed once 12 students had expressed interest in participating. We allowed 24 hours for poster placement at each canteen entrance.

Full-time students who are compliant with instructions from the research team were included. Students studying a nutrition major, suffering from eating disorders, and those who has metabolic diseases [20] were excluded. Without further assessment of participant nutrition literacy, 10 participants were randomly selected from 36 expression-of-interest using Excel spreadsheet random number function. Providing a sense of perceived fairness at this stage was important for facilitating authentic reporting for participants during ethnography [21].

## Training in the China cohort

In nutrition research, ethnography is unknown to Chinese university students. It was essential that the ethnographer familiarise participants in the China cohort with the rationale of a digital diet ethnography and expectations of participating in this study. Instead of collecting a series of diet diary with estimated nutrient intake, participants were encouraged to respond with a video recording of their eating occasion, including foods, daily activity, and the environment, regardless of their relevance to dietary intake. Much of the information was irrelevant to their dietary behaviour determinants. However, it was important to create an atmosphere as close as possible to their normal daily routine. When participants felt safe and relaxed, they were more likely to respond with authentic feelings and thoughts on their eating occasion, rather than providing predisposed answers that conform to clearly defined research objectives.

During the ethnography, two training sessions were provided by the ethnographer on 07 May 2022 and 08 Jun 2022, both via Tencent Meeting (session ID 176-220-468 and 757-653-709, respectively, Shenzhen, China) where all participants participated. The first training session took place one day before data collection commenced. In this session, the ethnographer introduced himself to the participants, explained the rationale behind this ethnography study, and demonstrated to the participants how Indeemo should be appropriately used for this study. Certain rules and expectations for information-rich responses were explained to the participant, too. The second training session was conducted after participants have finished all exams. This session facilitated effective responses after the students returned home when the semester ended. During this session, the researcher reflected on his observations during the first phase of digital diet ethnography, discussed common issues with participant responses, and explained how participants could feel more natural when they complete future tasks.

## Data Collection

### Duration

In the New Zealand cohort, data collection began on 18 Jul 2022 and continued for 3 months, finishing on 01 Nov 2022. In the China cohort, data was collected for 90 days from 08 May 2022 to 05 Aug 2022, inclusively. Both arms selected a 3-month period because the usual duration of diet ethnography were generally 3 months, with some situated at 6 to 18 months [22].

The China cohort data collection was divided into two tiers because HBMU commenced summer holidays since 08 Jun 2022. Tier 1 included responses from 08 May to 13 or 14 Jun 2022 where participants were boarding on campus in dormitories and were primarily exposed to the university food environment. Hence, the dietary behaviour dataset for tier 1 was named “on campus”. Two participants returned home on 13 Jun 2022 and eight participants returned home on 14 Jun 2022. Tier 2 included responses from 14 or 15 Jun 2022 to 05 Aug 2022 where participants were exposed to a wide variety of food environments, mainly home but also workplaces (hospitals), tourist cities/sites, and in a different university. Hence, the dietary behaviour dataset for tier 2 was named “at home”. These two tiers of data allowed a comparative analysis of dietary behaviour determinants among Chinese university students when they attended or were away from university.

### Type of Data Collected

In both arms, data was collected using Indeemo, a digital ethnography platform and mobile application. Prior to data collection, participants were assigned a series of daily tasks, each comprised of a set of questions which related to students’ dietary behaviours. The questions were designed to assist in contextualising eating occasions, by probing for information such as where the participant was, who they were with, what they were doing, what they were thinking, or how they were feeling. Participants were required to complete a total of four tasks on each calendar day: one each for breakfast, lunch, dinner, and snacks. Participants completed each task by creating one or more “responses”, which were equivalent to participant observations in conventional ethnography. When creating a response, participants could decide to take a photo, record a video, write a text description, or upload a combination of all three forms. In addition, the New Zealand ethnographer conducted interviews retrospectively on Indeemo to address unanswered questions.

### Monitoring Participant Responses

Keeping track of what the participant had been doing informed the ethnographer of any immediate actions that needed to be taken. Indeemo displayed a user interface for participants, and a “dashboard” interface for the ethnographers when logging in with a manager account. The “dashboard” portal collates all multimedia uploads from participants for the ethnographers to monitor responses in real time. A regular period of time, usually 09:30 – 10:30 a.m., was allocated each day where both ethnographers examined participant responses. When a task had not been completed within 24 hours since its deployment, the ethnographers prompted the participant for reasons why these tasks were missed and encouraged them to upload their responses. Participants sometimes could be “check-listing” the instructions when the ethnographers emphasised too much on following standard reporting procedures for eating occasion. As soon as an ethnographer identified biased responses, he talked to participants to resolve any issues preventing them from providing an authentic description of their eating occasion.

## Data Analysis

### Data Processing

Indeemo transcribed video responses in the New Zealand and China cohort into English and Chinese text, respectively. All final data used for analysis was in text form, including transcribed verbatim, text captions on photos, text-only responses, and interview notes. For each participant, a summary Microsoft Word document was generated from Indeemo to include all data mentioned above, with date and time stamps. Both ethnographers listened to all video responses and rectified any errors in the transcribed verbatim in the final summary document. All summary documents were then imported into Nvivo 14 (Lumivero, Denver, United States) for systematic coding.

### Mixed Grounded Theory

In this study, we employed a mixed grounded theory approach of deductive and inductive coding to analyse qualitative data. Grounded theory emphasises on the discovery of latent themes by inductive “open coding” whereas qualitative content analysis breaks down the data into units based on their manifest meanings and then use deductive themes to help organise themes [23]. We viewed and coded participant responses as the data collection process was happening, which was standard practice for grounded theory studies. More importantly, we used inductive-abductive reasoning for data analysis rather than trying to deductively “fit” our data with any existing theoretical framework [24]. It should be noted that we neither called for a “saturation stop” nor changed the anticipated type of data to collect, as a conventional grounded theory ethnography usually does [25]. Overall, our data analysis closely resembled grounded theory but incorporated certain qualitative content analysis practices.

Since a pre-determined theoretical framework was involved, our data analysis procedure followed the suggested steps for qualitative content analysis described by Terry et al. [26], including six phases as shown in **Supplementary Table 1**. Both ethnographers from the New Zealand and China cohort independently completed initial coding for their corresponding data and justified the codes to their supervisor. When disagreements arose, two researchers discussed the codes and participant responses to reach a consensus. In cases of non-consensus, another supervising researcher was invited to decide on the case.

**Supplementary Table 1. The 6 steps of qualitative data analysis and procedures performed.**

| Step | Procedure |
| --- | --- |
| Familiarisation | Read through all participant transcripts, clean data by removing duplicates and correcting erroneous scripts, understand the context of participants’ eating occasions, and familiarise with potential codes or themes. Write memos where appropriate. |
| Generating initial codes | Add codes to participants’ dietary behaviour, either in vivo or after interpreting the transcript. Iteratively read back and forth the data, identify codes that were less apparent at first time of coding. Write memos where appropriate. |
| Constructing themes | Group codes together to generate sub-themes based on their meaning. Write memos where appropriate. |
| Reviewing potential themes | Compare and contrast sub-themes, evaluate sub-themes in the context of both the ethnography data and theoretical framework. Write memos where appropriate. |
| Defining and naming themes | Group sub-themes together to form major themes, resolve overlapping themes and name major themes properly to reflect the meanings they encompass. Write memos where appropriate. |
| Producing the report | With the aid of memos written during data analysis, summarise the initial codes, sub-themes, and major themes concisely, provide justification with direct quotations when necessary, and explain the logical connection between codes or themes. |

## The Hawthorne Effect

The phenomenon by which research subjects diverting from their “normal” behaviour as a result of knowing to be observed, is often regarded as one of the most significant barriers for ethnographers to draw valid conclusions from their experiments, known as participant reactivity or Hawthorne effect [27, 28]. According to Muchinsky [29], the Hawthorne effect is temporary and could last for several days or up to 2 years. In this study, we took advantage of our feasibility study to inform our digital diet ethnography practices to avoid the Hawthorne effect. Olson & Verley [30] synthesised several important themes regarding the recommended precautions to take. Our countermeasures performed during the ethnographic study regarding each theme were summarised in **Supplementary Table 2**.

**Supplementary Table 2. Digital diet ethnography practices to avoid the Hawthorne effect.**

| Variable to consider | Countermeasures |
| --- | --- |
| Social norms | Participants were encouraged on their willingness of taking videos over responding with text or photos. |
| Dietary behaviour is complex/multiply determined | Using an open-ended task list, the participants can describe their eating occasion from any perspective. |
| Extraneous variables or experimental design flaws | No pre-defined variable was expected or requested from the participants. |
| Participant reactivity to experimental conditions | The researcher organised multiple online meetings and functioned as a participant-researcher to minimise the impact of the “experimental” atmosphere. ^*^ |
| Incentives affect behaviour | Participants are incentivised based on whether they could complete all tasks with satisfactory information of their eating occasion. |
| Measurement validity | The researcher posted videos as an exemplar for participants for them to understand what an authentic description of an eating occasion. |
| Supportive supervision | Every day the responses from participants were examined by the researcher and any issues with understanding their tasks will be reflected on. |

^*^Only applicable in the China cohort.

Among all practices, perhaps those minimising the participants’ feeling of being observed were the most effective. For example, the ethnographer in the China cohort created a group chat via the most popular mobile social networking application in China, WeChat (Tencent, Shenzhen, China), with all participants in the group. The ethnographer recorded every eating occasion as his own responses to each task, every day for 90 consecutive days, exactly as the participants were expected to do. He sent recorded videos, pictures, and/or text to the group chat where all participants had access to. When the participants became the “observer” of their ethnographer’s dietary behaviours, their responses exhibited natural settings. In addition, both arms collected data over a long period of time, allowing rapport and trust to be established between the ethnographer and the participants [31]. This was particularly helpful among the Chinese population, including university students, because they could easily be influenced by instruction from researchers and participation in replacements [32-34].

## References

1. Bhanye, J., et al., Doing urban research on ‘hard-to-reach’populations during the COVID-19 pandemic: advantages and ethical dilemmas using digital ethnography as a new alternative. Discover Global Society, 2024. **2**(1): p. 45.

2. Ahmed, S.T. and T. Roche, Making the connection: Examining the relationship between undergraduate students’ digital literacy and academic success in an English medium instruction (EMI) university. Education and Information Technologies, 2021. **26**(4): p. 4601-4620.

3. Jensen, L.X., et al., Digital ethnography in higher education teaching and learning—a methodological review. Higher Education, 2022. **84**(5): p. 1143-1162.

4. Berg, B.L. and H. Lune, Qualitative research methods for the social sciences. 8th ed. ed. 2012, Boston: Pearson.

5. Timonen, V., G. Foley, and C. Conlon, Challenges when using grounded theory: A pragmatic introduction to doing GT research. International journal of qualitative methods, 2018. **17**(1): p. 1609406918758086.

6. Edmonds, W.A. and T.D. Kennedy, An Applied Guide to Research Designs: Quantitative, Qualitative, and Mixed Methods. 2017, SAGE Publications, Inc: Thousand Oaks

Thousand Oaks, California.

7. Lubans, D.R., et al., Explaining dietary intake in adolescent girls from disadvantaged secondary schools. A test of Social Cognitive Theory. Appetite, 2012. **58**(2): p. 517-524.

8. Brückner, K., A. Emberger-Klein, and K. Menrad, How do emotions influence healthy food choice? Investigating an extended framework of the social-cognitive theory. British Food Journal, 2024. **126**(13): p. 486-503.

9. Suwannawong, P.R., et al., Ecological system theory and community participation to promote healthy food environments for obesity and non-communicable diseases prevention among school-age children. Public health nutrition, 2023. **26**(7): p. 1488-1500.

10. Stavitz, J., Understanding Micronutrient Access through the Lens of the Social Ecological Model: Exploring the Influence of Socioeconomic Factors—A Qualitative Exploration. Nutrients, 2024. **16**(11): p. 1757.

11. Glanz, K., L.E. Burke, and B.K. Rimer, Health behavior theories. Philosophies and theories for advanced nursing practice, 2011: p. 247-270.

12. Perron, N., Bronfenbrenner’s ecological systems theory. College student development: Applying theory to practice on the diverse campus, 2017. **197**(23): p. 1-10.

13. Timlin, D., et al., Are dietary interventions with a behaviour change theoretical framework effective in changing dietary patterns? A systematic review. BMC public health, 2020. **20**: p. 1-18.

14. Contento, I., Determinants of food choice and dietary change: Implications for nutrition education. Corrigan. L. Nutrition education: linking research, theory, and practice, 2016. **3**: p. 30-58.

15. Li, X., et al., How Does the University Food Environment Impact Student Dietary Behaviors? A Systematic Review. Frontiers in Nutrition, 2022. **9**.

16. Mays, N. and C. Pope, Quality in qualitative research. Qualitative research in health care, 2020: p. 211-233.

17. Li, X., et al., Assessing food availability and healthier options in an urban Chinese university: a case study using the Chinese Nutrition Environment Measurement Survey for Stores (C-NEMS-S). BMC Public Health, 2024. **24**(1): p. 15.

18. The University of Auckland. KEY STATISTICS 2020. 2020 [cited 2024 21 Nov]; Available from: <https://cdn.auckland.ac.nz/assets/auckland/about-us/our-ranking-and-reputation/key-statistics/key-stats-2020.pdf>.

19. Delormier, T., K.L. Frohlich, and L. Potvin, Food and eating as social practice–understanding eating patterns as social phenomena and implications for public health. Sociology of health & illness, 2009. **31**(2): p. 215-228.

20. Nyhan, W., B. Barshop, and P. Ozand, Atlas of metabolic diseases second edition. 2005: CRC Press.

21. AcSS, R.I., Research ethics in ethnography/anthropology. European commission, 2013.

22. Ottrey, E., J. Jong, and J. Porter, Ethnography in nutrition and dietetics research: a systematic review. Journal of the Academy of Nutrition and Dietetics, 2018. **118**(10): p. 1903-1942. e10.

23. Cho, J.Y. and E.-H. Lee, Reducing confusion about grounded theory and qualitative content analysis: Similarities and differences. Qualitative report, 2014. **19**(32).

24. Charmaz, K. and K. Henwood, Grounded theory methods for qualitative psychology. The SAGE handbook of qualitative research in psychology, 2017. **2**: p. 238e256.

25. Charmaz, K., A constructivist grounded theory analysis of losing and regaining a valued self. Five ways of doing qualitative analysis, 2011: p. 165-204.

26. Terry, G., et al., Thematic analysis. The SAGE handbook of qualitative research in psychology, 2017. **2**: p. 17-37.

27. Jiménez-Buedo, M., Reactivity in social scientific experiments: what is it and how is it different (and worse) than a Placebo effect? European Journal for Philosophy of Science, 2021. **11**(2): p. 42.

28. Goodwin, M.A., et al., The Hawthorne effect in direct observation research with physicians and patients. Journal of evaluation in clinical practice, 2017. **23**(6): p. 1322-1328.

29. Muchinsky, P.M., Psychology applied to work : an introduction to industrial and organizational psychology. 8th ed. 2006, Belmont, CA: Thomson/Wadsworth.

30. Olson, R., et al., What we teach students about the Hawthorne studies: A review of content within a sample of introductory IO and OB textbooks. The Industrial-Organizational Psychologist, 2004. **41**(3): p. 23-39.

31. Oswald, D., F. Sherratt, and S. Smith, Handling the Hawthorne effect: The challenges surrounding a participant observer. Review of social studies, 2014. **1**(1): p. 53-73.

32. Li, R., et al., Efficacy of a stannous fluoride dentifrice for relieving dentinal hypersensitivity in Chinese population: an 8-week randomized clinical trial. Clinical Oral Investigations, 2024. **28**(4): p. 1-11.

33. Chen, H. and Y. Yu, The impact of social-emotional learning: A meta-analysis in China. Frontiers in psychology, 2022. **13**: p. 1040522.

34. Bai, Y., et al., Impact of online computer assisted learning on education: Experimental evidence from economically vulnerable areas of China. Economics of Education Review, 2023. **94**: p. 102385.

# Additional file 2: COREQ (COnsolidated criteria for REporting Qualitative research) Checklist

| **Topic** | **Item**  **No.** | **Guide Questions/Description** | **Reported in Section** |
| --- | --- | --- | --- |
| **Domain 1: Research team and reﬂexivity** | | | |
| *Personal characteristics* | | | |
| Interviewer/facilitator | 1 | Digital ethnography tasks were facilitated and field data collection overseen by the two primary ethnographers: Xingbo (XL) for the China cohort and Nick (NA) for the New Zealand cohort. Both performed participant onboarding, training, and ongoing field engagement. | 2.3 Research Team Reflexivity |
| Credentials | 2 | XL is a PhD candidate (MSc in Laboratory Medicine). NA holds an MSc in Nutrition/Dietetics. Supervisory team credentials include AB (Associate Professor, PhD), ZL (Professor, PhD), and RR (Senior Lecturer, PhD). | 2.3 Research Team Reflexivity |
| Occupation | 3 | XL: PhD candidate (University of Auckland); NA: MSc student; AB: Associate Professor (UoA); ZL: Department Director (Hebei Medical University); RR: Senior Lecturer (UoA). | 2.3 Research Team Reflexivity |
| Gender | 4 | The two primary ethnographers (XL and NA) are male. Supervisory team gender composition is shown in author bylines. | 2.3 Research Team Reflexivity |
| Experience and training | 5 | Neither primary ethnographer had extensive prior formal ethnography experience before the project; both completed pilot feasibility work, received hands-on training and ongoing supervision from senior investigators, and participated in coder training and methodological feedback from external advisors. | 2.3 Research Team Reflexivity  2.9 Data analysis |
| *Relationship with participants* | | | |
| Relationship established | 6 | Yes. Rapport-building occurred through onboarding sessions (Zoom/Tencent Meeting), introductory group communications (Slack for NZ; WeChat for China) and preliminary feasibility contacts prior to sustained data collection. | 2.7 Recruitment  2.10 The Hawthorne Effect |
| Participant knowledge of the interviewer | 7 | Participants were informed of the researcher’s role, project aims, and a brief biography (motivation/background) during the introductory sessions and via the Participant Information Sheet. | 2.7 Recruitment  2.10 The Hawthorne Effect |
| Interviewer characteristics | 8 | Reflexivity statements detail the ethnographers’ backgrounds (training trajectory, cultural familiarity with Chinese and Western university food cultures), potential sources of bias, and mitigation steps (memo-ing, supervisor debriefs, bilingual back-checking). | 2.3 Research Team Reflexivity |
| **Domain 2: Study design** | | | |
| *Theoretical framework* | | | |
| Methodological orientation and Theory | 9 | The study used digital diet ethnography combined with a hybrid analytic orientation: grounded-theory informed open coding integrated with qualitative content analysis and a six-phase thematic approach (Terry et al.). Cohort-specific sensitising frameworks were used (Social Cognitive Theory/Bronfenbrenner for NZ; Contento for China). | 2.2 Theoretical Framework  2.9 Data analysis |
| *Participant selection* | | | |
| Sampling | 10 | Convenience sampling was used in both cohorts. China recruitment involved poster advertisement at canteens with random selection from the expression-of-interest pool to ensure perceived fairness across canteens; NZ recruitment used social media/flyers and self-selection. | 2.4 Study Setting  2.7 Recruitment |
| Method of approach | 11 | NZ: online channels, email, campus flyers and email onboarding. China: on-site canteen posters with QR contact, followed by remote onboarding (Tencent Meeting), and ongoing engagement via WeChat. Indeemo was used for task delivery in both cohorts. | 2.7 Recruitment  2.8 Data Collection |
| Sample size | 12 | There were 19 participants who participated and completed this study (NZ = 9; China = 10). | 2.7 Recruitment |
| Non-participation | 13 | One NZ participant withdrew prior to completing data collection and was replaced; there were no additional dropouts among the final cohorts. The single withdrawal was for personal/logistical reasons as described in Methods. | 2.7 Recruitment |
| *Setting* | | | |
| Setting of data collection | 14 | Data were collected remotely via Indeemo while participants were in naturalistic settings (university canteens, dormitories/flat kitchens, or family homes). The platform captured videos, photos and text for each eating occasion. | 2.4 Study Setting  2.8 Data Collection |
| Presence of non-  participants | 15 | No external researchers were present during participant recordings. Naturally occurring presence of roommates, partners, or family members during meal recordings was common and forms part of the dataset (documented in transcripts). | 2.8 Data Collection |
| Description of sample | 16 | All participants were full-time university students. NZ cohort: n=9 (age range 19–29; all female). China cohort: n=10 (6 female; age range 21-23). Additional background (living arrangement, field of study) is summarised in Results. | 3.1 Overview |
| *Data collection* | | | |
| Interview guide | 17 | Yes. An Indeemo task list and tutorial video functioned as semi-structured prompts; these were pilot tested during the Phase 1 feasibility study and refined before the main ethnography. | 2.8 Data Collection  Additional file 3 |
| Repeat interviews | 18 | Not applicable. The study collected continuous, daily multimodal ethnographic entries over 90 days rather than conducting scheduled repeat interviews. Ethnographers performed periodic check-ins. | N/A |
| Audio/visual recording | 19 | Yes. Participants submitted video recordings, photos with captions, and short text reflections using the Indeemo platform for each eating occasion. | 2.8 Data Collection |
| Field notes | 20 | Yes. Ethnographers maintained daily field memos and analytic notes which informed coding, memo-ing, and reflexive practice. These memos are referenced in Methods and stored in Supplementary materials. | 2.8 Data Collection |
| Duration | 21 | Data collection spanned 90 consecutive days (three months) for each cohort. | 3.1 Overview |
| Data saturation | 22 | Yes. Saturation was monitored by coding meal entries chronologically and tracking cumulative unique codes. Saturation was pragmatically defined as <1 new code per 10 successive meal entries plus no new substantive themes across two consecutive weeks. Using this criterion, approximately 95% of initial codes were identified by day 13–15, but all data were coded to allow exploratory theme development. | 2.9 Data Analysis |
| Transcripts returned | 23 | Full transcript return was not conducted due to high participant burden (large volume of short entries per participant). Participants were provided with a plain-language summary of findings and invited to comment via cohort communication channels. | 2.9 Data Analysis  4.8.2 Limitations |
| **Domain 3: Analysis and findings** | | | |
| *Data analysis* | | | |
| Number of data coders | 24 | Two primary coders (one ethnographer per cohort: XL for China; NA for NZ) completed initial coding. Coding was reviewed in consensus meetings with the supervisory team; unresolved disagreements were adjudicated by a senior investigator. | 2.9 Data Analysis |
| Description of the coding tree | 25 | Yes. The coding framework (major themes, subthemes, and exemplar initial codes) is summarised in the Results tables (Table 1; Table 2). | 2.9 Data Analysis |
| Derivation of themes | 26 | Themes were derived using a hybrid approach: inductive open coding produced initial codes and subthemes, which were iteratively compared and, where appropriate, mapped to cohort-specific sensitising frameworks (deductive elements). | 2.9 Data Analysis |
| Software | 27 | NVivo 14 was used for data organisation, coding and retrieval. | 2.9 Data Analysis |
| Participant checking | 28 | Participants were provided with a plain-language summary of major findings and invited to comment via group channels (WeChat/Slack); however, no formal transcript-level member checking or structured participant validation exercises were performed because of logistical burden. | N/A |
| *Reporting* | | | |
| Quotations presented | 29 | Yes. Selected illustrative quotations are presented throughout the Results to exemplify themes and are identified by cohort and participant code. Additional exemplars are available in Supplementary materials. | Table 2  3.3 China Cohort |
| Data and ﬁndings consistent | 30 | Yes. The Results are grounded in multimodal data and supported by exemplar quotations, which demonstrates clear linkage between raw data and analytic themes. | 3. Results |
| Clarity of major themes | 31 | Yes. Major themes are clearly presented for each cohort and synthesised in the cross-cohort comparison; Table 1 (NZ) and Table 2 (China merged) summarise the major themes and subthemes. | 3. Results  Table 1  Table 2 |
| Clarity of minor themes | 32 | Yes. Minor subthemes and less common or divergent cases are discussed in the Results narrative. | 3. Results |

Developed from: Tong A, Sainsbury P, Craig J. Consolidated criteria for reporting qualitative research (COREQ): a 32-item checklist for interviews and focus groups. *International Journal for Quality in Health Care*. 2007. Volume 19, Number 6: pp. 349 – 357

# Additional file 3: Indeemo task list for university student digital diet ethnography.

| **Entry Title** | **Entry Contents (English)** | **Entry Contents (Chinese)** |
| --- | --- | --- |
| Brief Introduction | Welcome to our research project! Thank you for taking part.  Over the next **3 months** we would like you to use **Indeemo** to complete a **daily diary** related to your food consumption. There is an ongoing task of recording your consumption moments (anything you eat/drink between waking up and going to sleep), which you can record in any format you wish, such as a photo and description, written text only or even a video.  Once you have read and understood each task and are ready to respond, **simply tap on the + button on this screen**, choose the type of response you wish (Video, Photo and/or Note) and follow the prompts on each screen. When recording videos, please ensure:   - That your device is always in landscape mode. - Videos can be around 3 minutes, if its longer just upload another one!   NOTE: because of the way that Indeemo tasks are managed, each task or meal must be completed with an entry. If for example you consumed breakfast, lunch, dinner and supper on one day, complete a normal entry for those tasks. For any tasks or meals where no food/ drink was consumed (in this example snack 1, snack 2, snack 3, snack 4) upload a black photo with no text description to signify that nothing was consumed for this task. | 欢迎您来到我们的课题，感谢您的参与。在接下来的3个月里，我们需要您使用Indeemo（手机APP）来完成每日膳食日记。这项研究的内容是记录你的饮食行为，包括摄入的任何食物及饮品，你可以用不同的方式记录，比如拍照片、文字描述、录制视频等。  Indeemo每天都会在固定时间节点向你推送膳食记录任务，当你阅读并了解每项任务后，只需点击屏幕上的“+”按钮，根据你想选择的回应类型（视频、照片和/或文字）按照屏幕上的提示逐步操作即可。  **特别提示**：录制视频时，请确保：  - 你的设备始终处于横向模式。  - 视频时长可以达到3分钟左右，如果视频比较长，分成多段视频上传即可。  由于Indeemo的任务管理方式，每项任务或每餐都必须填写晚餐。例如，如果你在一天内吃了早餐、午餐、晚餐和夜宵，请按正常流程完成这些任务。对于没有摄入食物或饮品的任务（比如没有吃早餐），上传一张空白照片即可。  请确保完成每项任务时你的回应包含任务描述中所要求的信息，并在其基础上尽可能更深入地聊一聊你认为哪些因素影响了你本次就餐所选择的食物类型。 |
| Task Title & Task Description 1 | **Breakfast (NOTE: DAILY TASK)**  What did you have for breakfast this morning?   - Where were you during this consumption moment? - What were you doing during this consumption moment? - What were you thinking during this consumption moment? - How were you feeling before, during and after this consumption moment?   Required responses:  ANY (video, text, photo) | 早餐（每日）  你今天早餐吃了什么？   - 本次就餐你在什么地点？ - 本次就餐时你是否在做其他的事情？ - 本次就餐时你是否与其他人共同就餐？ - 本次就餐之前、就餐时、就餐后你有什么感受？   回应类型：任意（视频、文字、照片） |
| Task Title & Task Description 2 | **Lunch (NOTE: DAILY TASK)**  What did you have for lunch this morning/afternoon?   - Where were you during this consumption moment? - What were you doing during this consumption moment? - What were you thinking during this consumption moment? - How were you feeling before, during and after this consumption moment?   Required responses:  ANY (video, text, photo) | 午餐（每日）  你今天午餐吃了什么？   - 本次就餐你在什么地点？ - 本次就餐时你是否在做其他的事情？ - 本次就餐时你是否与其他人共同就餐？ - 本次就餐之前、就餐时、就餐后你有什么感受？   回应类型：任意（视频、文字、照片） |
| Task Title & Task Description 3 | **Dinner (NOTE: DAILY TASK)**  What did you have for dinner this evening?   - Where were you during this consumption moment? - What were you doing during this consumption moment? - What were you thinking during this consumption moment? - How were you feeling before, during and after this consumption moment?   Required responses:  ANY (video, text, photo) | 晚餐（每日）  你今天晚餐吃了什么？   - 本次就餐你在什么地点？ - 本次就餐时你是否在做其他的事情？ - 本次就餐时你是否与其他人共同就餐？ - 本次就餐之前、就餐时、就餐后你有什么感受？   回应类型：任意（视频、文字、照片） |
| Task Title & Task Description 4 | **Night Snack (NOTE: DAILY TASK)**  What did you have for dinner this evening?   - Where were you during this consumption moment? - What were you doing during this consumption moment? - What were you thinking during this consumption moment? - How were you feeling before, during and after this consumption moment?   Required responses:  ANY (video, text, photo) | 夜宵（每日）  注：夜宵也包括在教室、宿舍等地点进食的零食、饮品等。  你今天夜宵吃了什么？   - 本次就餐你在什么地点？ - 本次就餐时你是否在做其他的事情？ - 本次就餐时你是否与其他人共同就餐？ - 本次就餐之前、就餐时、就餐后你有什么感受？   回应类型：任意（视频、文字、照片） |
| Task Title & Task Description 5 | **Interview questions**  Occasionally the researcher will ask additional questions about specific eating occasions in order to gain a deeper level of understanding to the factors that influenced what was being consumed during that specific eating occasion. This will be answered using a text response.  That's it! Thank you so much for taking part. As a thank you, we will send you a $50 supermarket voucher following your first week of Indeemo diary logging. | **访谈提问**  研究者会在不特定的时间询问一些关于某些饮食情景的附加问题，以便更深层次地了解影响该饮食情景消费的因素。你可以用文字回复。  研究完成后，我们将根据参与者在Indeemo完成任务的时长及质量给予现金或商品奖励，具体请查阅手册。以上是本研究的全部内容，再次感谢您的参与。 |
